# Optimization of Process Parameters for Laser Remelting Nickel Coating

**DOI:** 10.3390/mi17060646

**Published:** 2026-05-24

**Authors:** Hengzheng Li, Yangyang Li, Rui Zhan, Ruolan Shen, Xiaobo Song, Conghu Liu

**Affiliations:** 1 School of Mechanical and Electronic Engineering, Suzhou University, Suzhou 234000, China; 2 Anhui Lingzhou Technology Innovation Co., Ltd., Suzhou 234000, China; 3 School of Business Administration, Guangxi University, Nanning 530004, China

**Keywords:** laser remelting, nickel plating, surface morphology, wear resistance

## Abstract

Nickel coating exhibits excellent wear resistance. To further improve its surface performance, laser remelting technology was employed in this study to modify the surface of nickel coating. In this study, pulsed laser with a wavelength of 1064 nm, pulse duration of 5 ms and laser spot diameter of 2 mm was employed to conduct remelting strengthening treatment on the nickel-based coating deposited on pure copper substrate. The effects of laser frequency and energy density on the coating were investigated by characterizing the surface morphology, microhardness, three-dimensional topography, and wear resistance of the laser-remelted samples. The results indicate that laser frequency influences the surface properties mainly by changing the overlapping ratio of the remelted spots. Laser energy density affects the remelting zone and thereby modifies the surface characteristics of the sample. When the laser frequency is 10 Hz and the energy density is 165.87 J/mm^2^, the sample obtains favorable surface roughness and wear resistance.

## 1. Introduction

With the rapid development of modern manufacturing, aerospace, and marine engineering fields, components serving in harsh environments (such as high load [1], corrosion [2,3], and wear [4]) have increasingly stringent requirements for surface mechanical properties. Pure copper is widely used in electrical and thermal conduction components due to its excellent electrical and thermal conductivity, but its inherent low hardness and poor wear resistance limit its application in complex service scenarios. To address this defect, depositing a nickel-based coating on the pure copper surface has become a common surface modification technology, as nickel-based coatings possess good hardness, wear resistance, and corrosion resistance, which can effectively improve the service life of pure copper components [5,6].

However, the nickel-based coating prepared by conventional deposition methods (such as electroplating and thermal spraying) often has defects such as loose structure [7], poor bonding force with the substrate [8,9], and uneven component distribution [10], which severely restrict the further improvement in the surface comprehensive properties of pure copper components. Laser remelting uses high-energy laser beams to rapidly melt and solidify the surface layer of materials so as to refine the surface microstructure and eliminate internal pores and other defects [11,12,13]. As an advanced surface modification technology, it has been widely applied in the field of metal surface modification. In addition, when laser remelting is adopted to strengthen surface coatings, metallurgical bonding can be formed between coatings and substrates, which can significantly improve the interfacial bonding strength between them [14,15,16].

At present, various studies and engineering applications have been carried out on laser remelting technology. However, most existing studies mainly focus on the surface modification and strengthening of single metallic materials, while studies concerning laser remelting of dissimilar materials remain insufficient. Specifically, the influence mechanisms of laser remelting process parameters on the surface morphology, microhardness and surface roughness of nickel-based coatings have not been clarified, and systematic investigations on the property regulation mechanism of coatings are still lacking. There exist prominent differences in key physical parameters between copper and nickel, such as thermal conductivity, melting point and coefficient of thermal expansion. Accordingly, the laser remelting mechanism, process parameter optimization and surface property regulation rules of nickel-based coatings deposited on pure copper substrates are far more complicated than those of surface modification for single metallic materials.

In view of the above-mentioned problems, this study selects nickel-based coatings on pure copper substrates as the research object, and conducts remelting strengthening treatment by using pulsed laser with a wavelength of 1064 nm, pulse duration of 5 ms and laser spot diameter of 2 mm. The purpose of this research is to reveal the influence mechanism of laser process parameters on the surface morphology and mechanical properties of nickel-based coatings on pure copper substrates, optimize the laser remelting process, and provide technical references for the engineering application and performance enhancement in laser remelting technology in material coating modification.

## 2. Experimental

### 2.1. Experimental Materials

**Figure 1:**
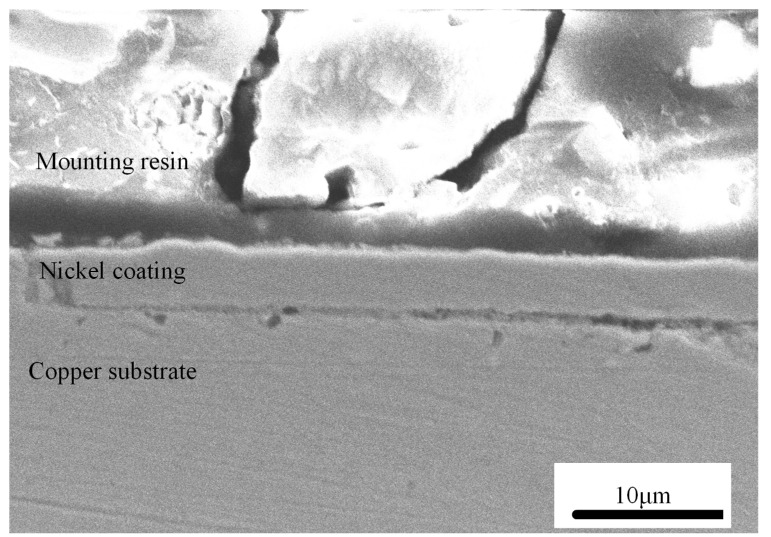
The cross-section of the nickel-plated sample.

The experiment used a purple copper plate (T2) with dimensions of 50 mm × 30 mm × 2 mm as the substrate. The composition of the copper plate is shown in Table 1. Before the experiment, the sample was cleaned with detergent to remove any oil stains on its surface. Then, the sample was polished to remove the surface oxide layer and fatigue layer. Finally, it was rinsed with deionized water and dried for later use. A constant temperature magnetic stirrer was used to nickel coat the copper plate (T2), with a plating solution temperature of 80 ± 2 °C and a pH of 4.0–4.5. The main components of the plating solution are listed in Table 2. The immersion plating time of the sample was 2 h, and the thickness of the obtained coating was approximately 5 μm. The cross-section of the nickel-plated sample is shown in Figure 1.

### 2.2. Equipment and Characterization

**Figure 2:**
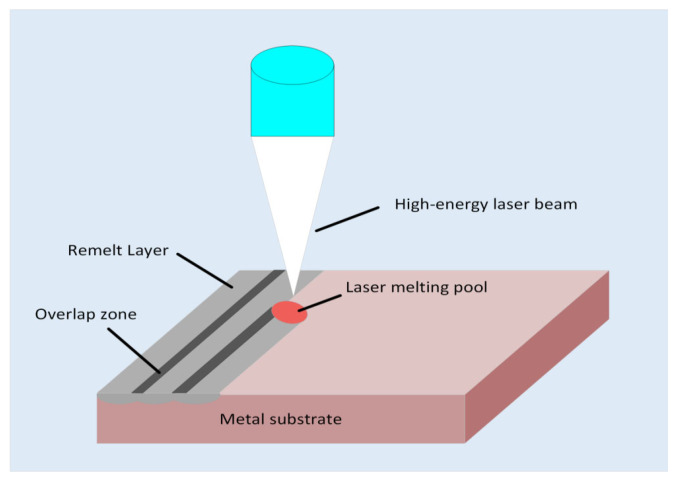
Schematic illustration of the laser surface remelting procedure.

Surface treatment of the sample was performed by employing a pulsed laser device (model: TY-LFS-500, manufactured by Wuhan Tianyi Laser Equipment Co., Ltd., Wuhan, Hubei, China). A Nd:YAG solid-state laser was employed in this equipment, with a laser wavelength of 1064 nm, a laser pulse duration of 5 ms, and a spot diameter of 2 mm. With the above parameters kept unchanged, the laser frequency was set to 5, 10 and 15 Hz, respectively, and the laser power was set to 75, 100, 125 and 150 W (corresponding to laser energy densities of 99.52 J/mm^2^, 132.69 J/mm^2^, 165.87 J/mm^2^ and 199.04 J/mm^2^). Surface strengthening treatment was carried out on the samples under these conditions. The device was operated at a feeding rate of 5 mm/min, and the scanning spacing was set to 0.5 mm. The schematic illustration of the laser surface remelting procedure is presented in Figure 2.

The surface morphology of the treated sample was observed and characterized using a scanning electron microscope (SEM, model S4800, Hitachi Ltd., Tokyo, Tokyo Metropolis, Japan) under an accelerating voltage of 20 kV. A laser confocal 3D measuring instrument (model SM-5100, produced by Sixian Optoelectronics Technology (Shanghai) Co., Ltd., Shanghai, China) was utilized to reconstruct the three-dimensional surface topography of the sample and measure its surface roughness, where the final roughness value was taken as the average of three parallel measurements.

Wear resistance tests were conducted on the samples with a high-speed reciprocating friction and wear tester (model HSR-2M, Lanzhou Zhongke Kaihua Technology Development Co., Ltd., Lanzhou, Gansu, China). The friction pair adopted was a GCr15 alloy ball with a diameter of 4 mm. The test parameters were set as follows: a friction load of 100 g, a friction duration of 5 min, a reciprocating stroke of 5 mm, and a reciprocating frequency of 500 cycles per minute.

## 3. Results and Discussion

### 3.1. The Effect of Process Parameters on the Surface Morphology

#### 3.1.1. The Effect of Laser Frequency on Surface Morphology

**Figure 3:**
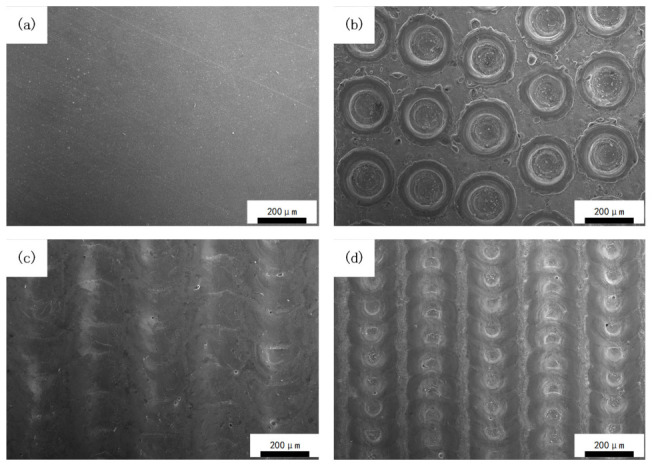
SEM images of specimens at various laser frequencies: (**a**) blank, (**b**) 5 Hz, (**c**) 10 Hz, (**d**) 15 Hz.

Figure 3 shows SEM images of specimens at various laser frequencies with a fixed laser energy density of 165.87 J/mm^2^. It can be seen from the figure that a smooth nickel coating is obtained on the sample surface after electroless nickel plating. At a laser frequency of 5 Hz (Figure 3b), discrete circular remelted spots appear on the sample surface. The center of the remelted spot is concave, while the surrounding area is raised, with obvious material flow traces. Although the remelted spots are distributed linearly, the adjacent remelted spots do not overlap. Therefore, a complete remelted layer is not formed on the sample surface. At a laser frequency of 10 Hz (Figure 3c), the circular remelted spots overlap each other. The overlapping area accounts for about 40% of the total remelted area, showing a fish-scale-like structure. Due to the overlapping of remelted spots, there are no obvious melting pits on the surface, and the remelted layer is fully covered. When the laser frequency is further increased to 15 Hz (Figure 3d), the surface exhibits a bamboo-like structure with obvious pits. In addition, band-like elevations exist between adjacent columns, and distinct band-like pits appear on the remelted layer.

The above variations in SEM images can be attributed to the small number of remelted spots generated per unit time at a low laser frequency. At a laser frequency of 5 Hz, the distribution density of the remelted spots is relatively low, which fails to coordinate with the feeding rate of the laser device, thus giving rise to a discontinuous remelted layer on the sample surface. As the laser frequency is raised to 10 Hz, the quantity of laser-acted spots per unit time becomes appropriate, which can effectively cover the scanning trajectory and guarantee a reasonable overlapping rate between adjacent remelting regions. When the laser frequency is excessively high, an excessive number of remelted spots are generated per unit time, causing the already overlapped remelted spots to be irradiated again. Therefore, obvious bamboo-like protrusions appear on the sample surface. The uplifts between adjacent columns are caused by the superposition of multiple material flows.

#### 3.1.2. The Effect of Laser Energy Density on Surface Morphology

**Figure 4:**
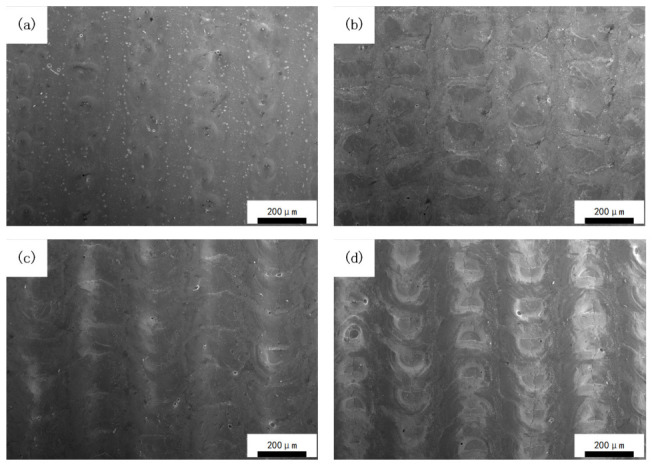
SEM images of specimens at various laser energy densities: (**a**) 99.52 J/mm^2^, (**b**) 132.69 J/mm^2^, (**c**) 165.87 J/mm^2^, (**d**) 199.04 J/mm^2^.

Figure 4 shows the SEM images of specimens at various laser energy densities at a fixed laser frequency of 10 Hz. By comparing Figure 4a–d, it can be seen that the strip-like processing traces after laser remelting gradually become more prominent with increasing laser energy density. At an energy density of 99.52 J/mm^2^ (Figure 4a), only slight pits form at the center of the remelted spots due to the low laser energy input. At this stage, the temperature rise in the remelted spots is relatively low, and the material fluidity is insufficient; so, the remelted spots cannot overlap. With a continuous increase in laser energy density, the temperature of the remelted spots rises gradually, the melting area and material fluidity are both improved, and the overlapping of remelted spots is finally achieved. At a laser energy density of 199.04 J/mm^2^, large bamboo-like melting pits appear on the surface, and local coating breakdown occurs.

It is worth noting that small cracks appear on the sample surface at laser energy densities of 165.87 J/mm^2^ and 199.04 J/mm^2^. The size and number of cracks increase with increasing laser energy density. The main reason for this phenomenon is the stress difference generated on the sample surface after laser remelting. During the remelting process, the molten material flows from the center of the remelted spot to the periphery, resulting in a thickness difference between the edge and the center of the remelted spot. During rapid solidification, this thickness difference induces significant residual stress, leading to crack formation in the sample [17,18,19]. In addition, the difference in thermal expansion coefficient between the nickel coating and the copper substrate is also an important factor.

### 3.2. The Effect of Process Parameters on Microhardness

#### 3.2.1. The Effect of Laser Frequency on Microhardness

**Figure 5:**
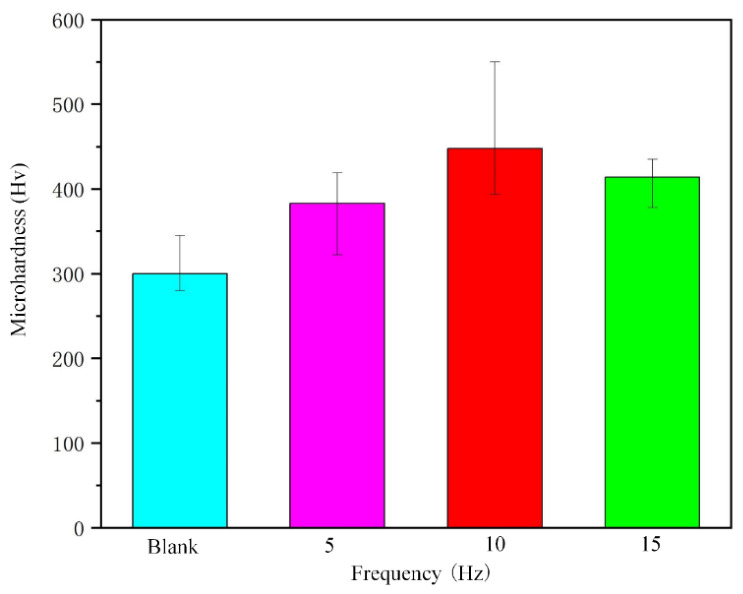
Microhardness of samples with different laser frequencies.

The microhardness of samples with different laser frequencies is presented in Figure 5. It is evident that the microhardness of the sample subjected to electroless nickel plating is roughly 300 Hv. Following laser remelting treatment, there is a remarkable enhancement in surface microhardness. Specifically, at pulse frequencies of 5 Hz, 10 Hz, and 15 Hz, surface microhardness reaches 383 Hv, 448 Hv, and 414 Hv, respectively. With the increase in laser frequency, microhardness exhibits a parabolic variation tendency.

Among the investigated parameters, the pulse frequency of 10 Hz delivers the strongest surface strengthening performance. The main reason is that at a low laser frequency, the surface remelted layer is not fully covered, and the unprocessed regions lower the overall average microhardness. An excessively high laser frequency gives rise to repeated remelting within the same area, resulting in the formation of abundant structural defects including pores and slag inclusions. Such defects impose an adverse effect on the enhancement in microhardness and consequently give rise to a reduction in hardness.

#### 3.2.2. The Effect of Laser Energy Density on Microhardness

**Figure 6:**
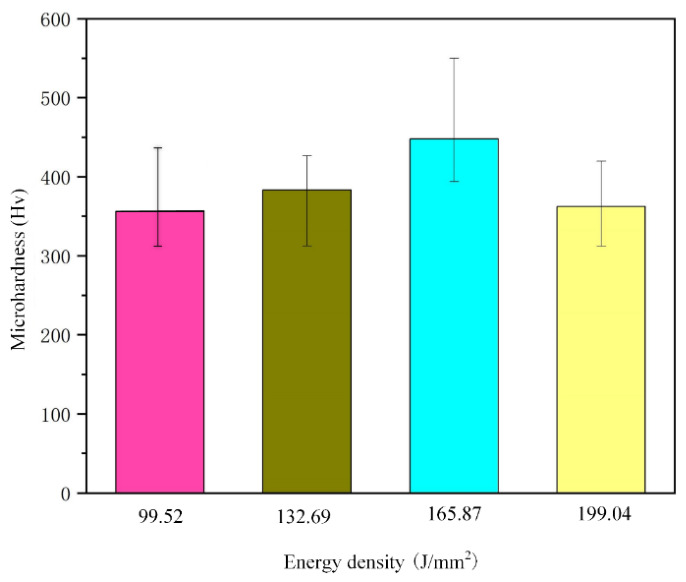
Microhardness of samples with different laser energy densities.

The microhardness variations in specimens with different laser energy densities are illustrated in Figure 6. The data indicate that when the laser energy density is below 165.87 J/mm^2^, microhardness increases gradually with rising energy density. This is related to the influence of laser energy density on the melting state of the remelted spots. As shown in the previous surface morphology analysis, at low energy densities, only the central region of the remelted spots is effectively melted, while the surrounding areas are not significantly melted. With increasing laser energy density, the melting area of the remelted spots gradually expands and favorable overlapping is achieved. Thus, within this parameter range, microhardness increases with energy density. As the laser energy density rises to 199.04 J/mm^2^, the excessive energy input induces severe sparking and material splashing on the specimen surface. This leads to a higher density of defects, including pores and inclusions, within the remelted layer, thereby causing a decline in microhardness.

### 3.3. The Effect of Process Parameters on Surface Roughness

#### 3.3.1. The Effect of Laser Frequency on Surface Roughness

**Figure 7:**
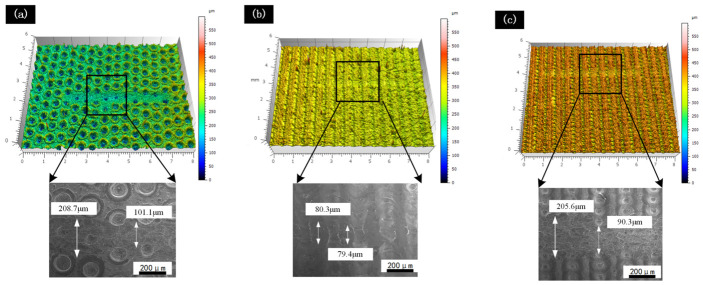
3D morphologies and wear marks of samples with different laser frequencies: (**a**) 5 Hz, (**b**) 10 Hz, (**c**) 15 Hz.

**Figure 8:**
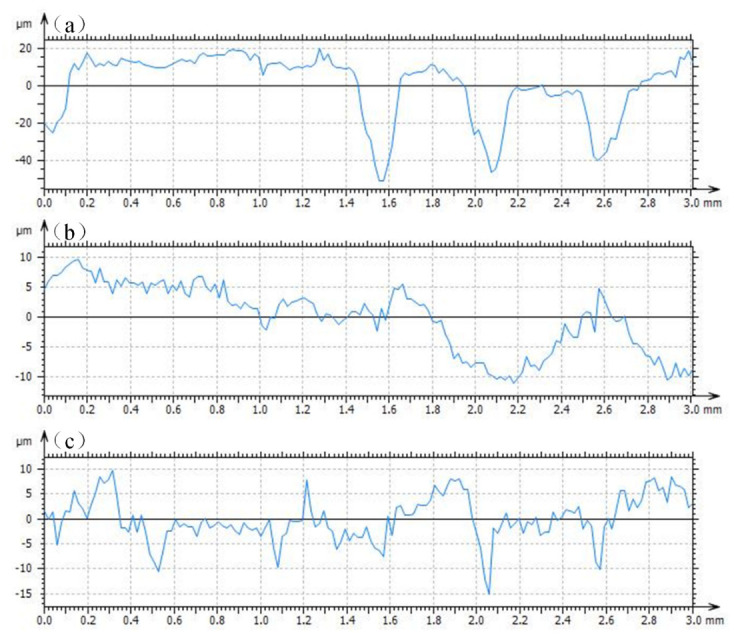
Surface profiles direction under different frequency parameters: (**a**) 5 Hz, (**b**) 10 Hz, (**c**) 15 Hz.

Figure 7 shows the three-dimensional morphologies and wear marks of samples under different laser frequencies. As shown in Figure 7a, at 5 Hz, numerous isolated remelted spots form on the sample surface. Each remelted spot is depressed in the center and raised at the periphery, with a depression depth of approximately 100 μm and a protrusion height of about 80 μm. When the laser frequency increases to 10 Hz, the remelted spots along the feeding direction overlap, forming a plow-groove-like structure with a pit depth of about 30 μm and an edge protrusion height of about 20 μm. As the pulse frequency further increases, the groove-like structure remains, but its depth increases to approximately 60 μm and the edge protrusion height rises to 50 μm. Figure 8 shows the surface profiles perpendicular to the laser feeding direction under different frequency parameters. The profile of the specimen is basically consistent with its 3D morphologies in Figure 7.

Table 3 presents the surface roughness of the specimens under different laser frequencies. It can be seen from the table that the sample without laser remelting treatment exhibits favorable surface roughness, with an average surface roughness value of 2.45 μm. The overall surface roughness exhibits a U-shaped trend with increasing laser frequency. When the pulse frequency of laser is 10 Hz, the surface roughness of the specimen reaches its minimum value of 13.23 μm. The above variation in surface roughness is mainly caused by the influence of laser frequency on the overlapping effect of remelted spots, which has been analyzed in detail previously and will not be repeated here.

By comparing the SEM images of the wear marks, the average widths of the wear marks are 154.4 μm, 79.9 μm, and 170.4 μm as the laser frequency increases from 5 Hz to 15 Hz. With increasing laser frequency, the wear loss first decreases and then increases, which is consistent with the microhardness results. The uneven width of the wear marks in the images may be related to the thickness distribution at the overlapping edges of the remelted spots.

#### 3.3.2. The Effect of Laser Energy Density on Surface Roughness

**Figure 9:**
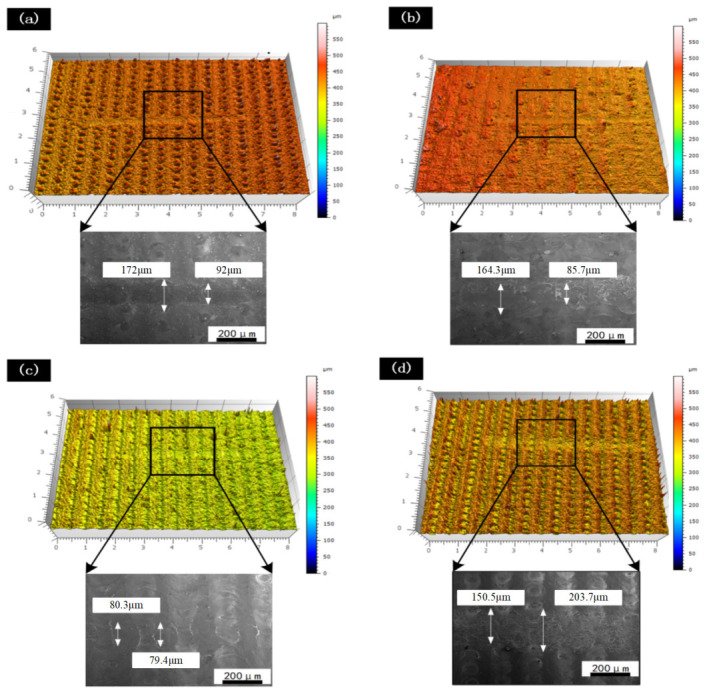
3D morphologies and wear tracks of specimens processed at various laser energy densities: (**a**) 99.52 J/mm^2^, (**b**) 132.69 J/mm^2^, (**c**) 165.87 J/mm^2^ (**d**) 199.04 J/mm^2^.

**Figure 10:**
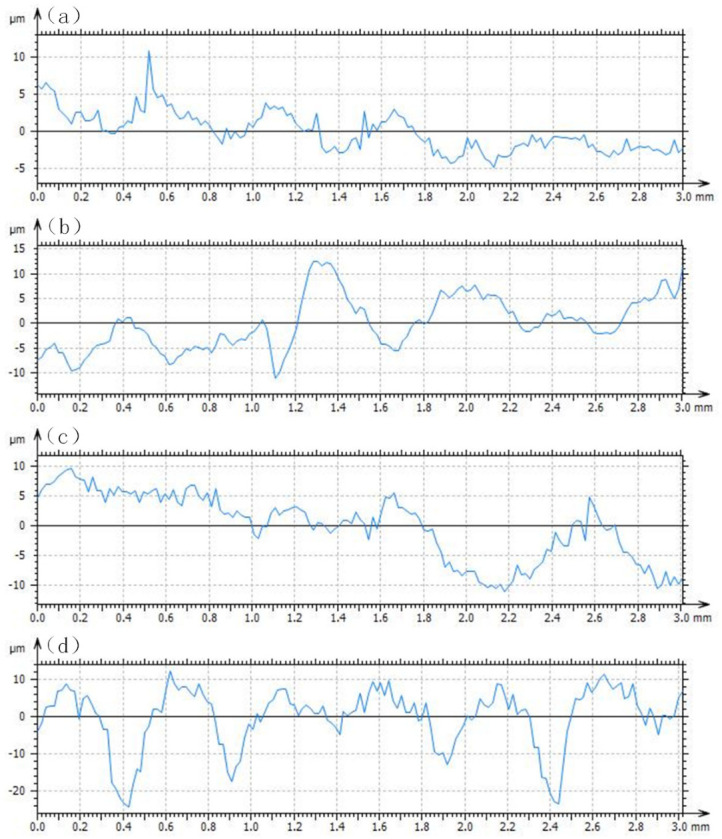
Surface profiles under different laser energy densities: (**a**) 99.52 J/mm^2^, (**b**) 132.69 J/mm^2^, (**c**) 165.87 J/mm^2^ (**d**) 199.04 J/mm^2^.

Figure 9 presents the 3D surface morphologies and wear tracks of specimens processed at various laser energy densities. Figure 10 shows the surface profiles perpendicular to the laser feeding direction under different laser energy density. From the 3D topography, a band-like convex hull structure appears on the surface at 99.52 J/mm^2^, with a convex hull diameter of about 150 μm and height of about 50 μm, and point-like pores at the center. When the laser energy density increases to 132.69 J/mm^2^, only a few convex hulls remain. Upon further increasing the energy density, the convex structures gradually vanish, leaving behind groove-like morphologies on the surface.

This is because at low laser energy density, the temperature rise in the remelted spots is low and the material fluidity is poor. After laser impact, the material cannot flow outward in time and solidifies rapidly. At higher energy density, the temperature of the remelted spots is higher and the material fluidity is better, allowing for sufficient flow before solidification.

Table 4 lists the surface roughness values under different laser energy densities. The variation trend of surface roughness is consistent with the SEM surface morphology and 3D topography results. SEM images of the wear marks show that with increasing laser energy density, the average wear widths are 122.3 μm, 105 μm, 84.3 μm, and 177.1 μm, respectively. The wear resistance first improves and then decreases, which is consistent with the microhardness trend. This phenomenon is related to the effect of laser energy density on the melting state of the remelted spots, as discussed in detail earlier.

## 4. Conclusions

This study systematically investigated the process optimization of laser remelting for electroless nickel coatings on pure copper substrates. The effects of laser frequency and energy density on surface morphology, microhardness, surface roughness, and wear resistance were analyzed, and the optimal process parameters were determined. The main conclusions are drawn as follows:

(1) Laser frequency mainly affects surface quality by regulating the overlapping ratio of remelted spots. At 5 Hz, the remelted spots are discrete and fail to form a continuous remelted layer. At 15 Hz, excessive overlapping leads to bamboo-like structures, pits, and defects. In contrast, 10 Hz yields uniform fish-scale-like morphology with appropriate overlapping (about 40%), ensuring a dense and complete remelted layer.
(2) Laser energy density determines the melting degree and fluidity of the coating. Low energy density (99.52 J/mm^2^) results in insufficient melting and poor overlapping. Excessively high energy density (199.04 J/mm^2^) causes severe melting, splashing, microcracks, and structural defects. The energy density of 165.87 J/mm^2^ achieves balanced melting, uniform structure, and favorable surface integrity.
(3) Laser remelting significantly refines the microstructure and improves the mechanical properties of nickel coatings. Microhardness first increases and then decreases with the rise in laser frequency and energy density, showing a parabolic trend. Surface roughness exhibits a U-shaped variation with laser frequency and gradually rises with excessive energy input. The wear resistance is highly consistent with microhardness, indicating that higher hardness corresponds to better wear resistance.
(4) Under the optimized parameters of 10 Hz and 165.87 J/mm^2^, the nickel coating achieves the best comprehensive performance: surface roughness Sa is 13.23 μm, microhardness reaches 448 Hv, and the wear track width is minimized. The coating presents uniform, dense, and defect-free surface morphology with outstanding wear resistance.

## Figures and Tables

**Table 1:** The composition of the copper plate.

Element	Ag	Zn	Pb	Fe	P	Cu
Mass ratio (%)	≤0.015	≤0.005	≤0.005	≤0.005	≤0.005	Bal.

**Table 2:** Components of the plating solution.

Components	g/L
Nickel sulfate	25
Sodium hypophosphite	26
Sodium acetate	10
Citric acid	14
Lactic acid	15

**Table 3:** Surface roughness (Sa) of samples with different laser frequencies.

Frequencies (Hz)	Sa1 (μm)	Sa2 (μm)	Sa3 (μm)	Average (μm)
Blank	2.43	2.44	2.48	2.45
5	18.53	19.31	18.34	18.72
10	12.50	13.95	13.25	13.23
15	17.61	15.58	15.34	16.18

**Table 4:** Surface roughness (Sa) of samples with different laser energy densities.

Energy Densities (J/mm^2^)	Sa1 (μm)	Sa2 (μm)	Sa3 (μm)	Average (μm)
99.52	5.21	6.96	5.43	5.87
132.69	10.22	8.71	9.93	9.62
165.87	12.50	13.95	13.25	13.23
199.04	14.97	14.51	14.66	14.71

## Data Availability

The original contributions presented in this study are included in the article. Further inquiries can be directed to the corresponding authors.
